# Incidence and prevalence of pulmonary tuberculosis among patients with type 2 diabetes mellitus: a systematic review and meta-analysis

**DOI:** 10.1080/07853890.2022.2085318

**Published:** 2022-06-15

**Authors:** Qian Wu, Yang Liu, Yu-Bo Ma, Kui Liu, Song-Hua Chen

**Affiliations:** a Department of Tuberculosis Control and Prevention, Zhejiang Provincial Center for Disease Control and Prevention, Hangzhou, China; b Department of Infectious Diseases Control and Prevention, Jiaxing Center for Disease Control and Prevention, Jiaxing, China; c Department of Epidemiology and Biostatistics, School of Public Health, Anhui Medical University, Hefei, China

**Keywords:** Incidence, prevalence, pulmonary tuberculosis, type 2 diabetes mellitus, burden

## Abstract

**Background:**

The epidemic of type 2 diabetes mellitus (T2DM) poses a great challenge to pulmonary tuberculosis (PTB) control. However, the incidence and prevalence of PTB among T2DM patients has not been fully determined. This meta-analysis aimed to provide the estimation on the global incidence and prevalence of PTB among T2DM patients (T2DM-PTB).

**Methods:**

Online databases including Web of Science, PubMed, China National Knowledge Infrastructure and Cochrane Library were searched for all relevant studies that reported the incidence or prevalence of T2DM-PTB through 31 January 2022. Pooled incidence and prevalence of T2DM-PTB with 95% confidence interval (CI) was estimated by the random-effect model. All statistical analyses were performed using R software.

**Results:**

A total of 24 studies (14 cohort studies, 10 cross-sectional studies) were included. The pooled incidence and prevalence of T2DM-PTB were 129.89 per 100,000 person-years (95% confidence interval (CI): 97.55–172.95) and 511.19 per 100,000 (95% CI: 375.94–695.09), respectively. Subgroup analyses identified that the incidence of T2DM-PTB was significantly higher in Asia (187.20 per 100,000 person-years, 95% CI: 147.76–237.17), in countries with a high TB burden (172.04 per 100,000 person-years, 95% CI: 122.98–240.68) and in studies whose data collection ended before 2011 (219.81 per 100,000 person-years, 95% CI: 176.15–274.28), but lower in studies using International Classification of Diseases-10 codes (73.75 per 100,000 person-years, 95% CI: 40.92–132.91). The prevalence of T2DM-PTB was significantly higher in countries with a high TB burden (692.15 per 100,000, 95% CI: 468.75–1022.04), but lower in Europe (105.01 per 100,000, 95% CI: 72.55–151.98).

**Conclusions:**

This systematic review and meta-analysis suggests high global incidence and prevalence of PTB among T2DM patients, underlining the necessity of more preventive interventions among T2DM patients especially in countries with a high TB-burden.

**Key messages**
- A total of 24 studies (14 cohort studies, 10 cross-sectional studies) containing 2,569,451 T2DM patients were included in this meta-analysis.
- The pooled incidence and prevalence of T2DM-PTB are 129.89 per 100,000 person-years (95% CI: 97.55–172.95) and 511.19 per 100,000 (95% CI: 375.94–695.09) respectively.
- The incidence of T2DM-PTB was significantly higher in Asia, in countries with a high TB burden and in studies whose data collection ended before 2011, but lower in studies using International Classification of Diseases-10 codes.
- The prevalence of T2DM-PTB was significantly higher in countries with a high TB-burden, but lower in Europe.

## Introduction

As a major global health problem, tuberculosis (TB) is a serious chronic infectious disease caused by *Mycobacterium tuberculosis* (MTB). TB occurs mostly in the lungs, leading to pulmonary TB (PTB). The 2021 Global TB reported that there were approximately 9.87 million new TB patients worldwide with an incidence rate of 127 per 100,000 in 2020 [1]. Although substantial efforts have contributed to the decline in the global TB epidemic, the pace of progress needs to be speed up to curb TB burden and achieve goals of WHO's “End TB strategy”.

Diabetes mellitus (DM) is a group of metabolic diseases characterized by hyperglycaemia, which is known to be an important predisposing factor in the development of PTB. The incidence of DM is increasing rapidly. In 2019, the number of DM patients has exceeded 460 million, which is expected to reach 700 million by 2045 [2]. Type 2 diabetes mellitus (T2DM) accounts for almost 90% of DM that caused by a combination of resistance to insulin action and an inadequate compensatory insulin secretory response [3]. T2DM frequently causes adverse microvascular and macrovascular comorbidities [4], such as chronic kidney disease, hypertension, myocardial infarction. A previous meta-analysis demonstrated that DM patients have two- to four-fold increased risk of active TB in comparison to the non-DM population [5], which may be related to the impaired innate and adaptive immune system caused by the long-term poor glycemic control. Additionally, the rates of treatment failure and death of PTB patients with concomitant DM are higher [6]. Therefore, it highlights the importance to understand the incidence and prevalence of T2DM-PTB co-morbidity, especially in high TB-burden countries.

To the best of our knowledge, a growing body of research have explored the mutual relationship of these two diseases and paid more attention to the bi-directional screenings of TB and DM [7]. Risk factors have been determined for PTB in T2DM patients previously [8–10]. However, current evidence from epidemiologic studies has been limited. Besides, the global incidence and prevalence of PTB among T2DM patients has not been fully determined. In order to implement comprehensive prevention strategy, it is imperative to conduct a systematic review and meta-analysis to review the current knowledge about the incidence and prevalence, and associated risk factors of PTB among patients with T2DM.

## Methods

### Search strategy

This meta-analysis followed the guidelines of Preferred Reporting Items for Systematic Reviews and Meta-Analyses statement. A literature search was conducted through electronic and manual searches on Web of Science, PubMed, China National Knowledge Infrastructure (CNKI) and Cochrane Library to identify all relevant studies published from their inception to 31 January 2022. The following keywords included: (“tuberculosis” or “TB” or “pulmonary tuberculosis” or “PTB”) and (“diabetes mellitus” or “DM” or “type 2 diabetes mellitus” or “T2DM”) without restriction to regions and languages. The bibliographic database searches were supplemented by screening the reference lists of all relevant publications. All search records were inserted into EndNote X8 software.

### Eligibility criteria

We included eligible observational studies (cohort and cross-sectional studies) that reported the incidence and/or prevalence of PTB in patients with T2DM or provided enough data (the number of T2DM-PTB cases and person-years of follow up) to compute the estimates. For studies with overlap population, only the study with the latest information or larger sample size was enrolled.

### Exclusion criteria

The exclusion criteria for the studies included in this meta-analysis were as follows: (1) the full text could not be retrieved, (2) studies were performed in animals, (3) data were insufficient for analysis, (4) the diagnostic criteria of T2DM were not clearly reported, (5) studies were published as reviews, meta-analysis, case series with small sample size (<50), meeting abstracts or editorials.

### Data extraction and quality assessment of included studies

Two investigators (Qian Wu and Yang Liu) independently screened the titles and abstracts of all articles for eligibility, and extracted the following data of each eligible study: first author’s name, publication year, the prevalence of T2DM-PTB (or number of T2DM and T2DM-PTB cases), the incidence of T2DM-PTB (or number of T2DM and T2DM-PTB cases, person-years of follow up), study period, study design, region, TB burden of study countries, definitions or diagnostic criteria for PTB. Quality of cohort studies was assessed using Newcastle-Ottawa quality assessment scale (NOS), with a score of ≥5 out of 10 considered as high-quality score [11]. Risk of bias in prevalence studies were assessed by using Hoy’s risk of bias tool [12]. Any disagreement between the researchers was resolved through discussion. The corresponding authors were contacted by e-mail for additional data.

### Statistical analysis

The incidence and prevalence of PTB among T2DM patients was reported with 95% confidence intervals (CIs) and presented in the forest plot. For cohort studies, T2DM-PTB incidence should be corrected for the total follow-up time and expressed as cases per 100,000 person-years. T2DM-PTB prevalence should be expressed as cases per 100,000. *I*^2^ statistic was used to quantify the effect of heterogeneity. In the case of significantly between-study heterogeneity (*I*^2^ value > 50% and the *p* value for Cochrane Q test <0 .10), the random-effects model was selected to compute the pooled estimate of prevalence [13]. Otherwise, the fixed-effect model was used. To identify the possible source of heterogeneity, we conducted subgroup analysis stratified by region, the TB burden of study countries, study design, diagnostic criteria for PTB and ending year of data collection. In addition, the visual inspection funnel plot with Egger’s linear regression test [14] and Begg’s rank correlation test [15] were used to assess potential publication biases. In order to assess the stability and reliability of the estimates, sensitivity analysis was conducted by iteratively removing one study from the meta-analysis. For all tests, *p* < 0.05 was deemed to be statistically significant. All statistical analyses were conducted using R software (version 3.6.1; http://www.R-project.org) with the “meta” package.

## Results

### Search results

**Figure 1.**
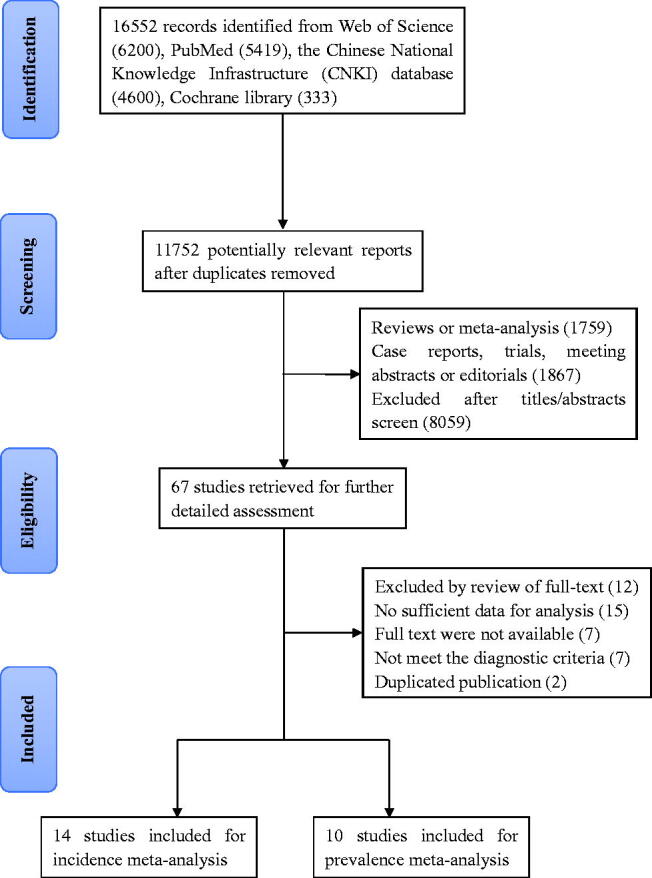
Flow chart of study selection.

After the comprehensive literature search, we identified 16,552 citations from Web of Science (6200), PubMed (5419), CNKI database (4600), Cochrane library (333). A total of 11,752 records remained after duplicates were excluded. After screening titles and abstracts, 67 eligible studies were retrieved. Finally, 43 studies were excluded accordingly and 24 studies [16–39] were included in this systematic review with meta-analysis (Figure 1). A total of 2,569,451 patients with T2DM were included, out of which 12,642 patients were T2DM-PTB patients. There were 14 cohort studies and 10 cross-sectional studies. All of these included studies were conducted from 1998 to 2018. The sample size of these studies ranged from 630 to 840,899. In terms of study region, 19 were from Asia (79.17%), 3 from Africa (12.50%) and 2 from Europe (8.33%). The majority of the included cohort studies were of moderate or high quality. The overall risk of bias of the prevalence studies was low in 7 (29.17%) studies, moderate in 12 (50%) studies and high in 5 (20.83%) studies.

### T2DM-PTB incidence

**Figure 2.**
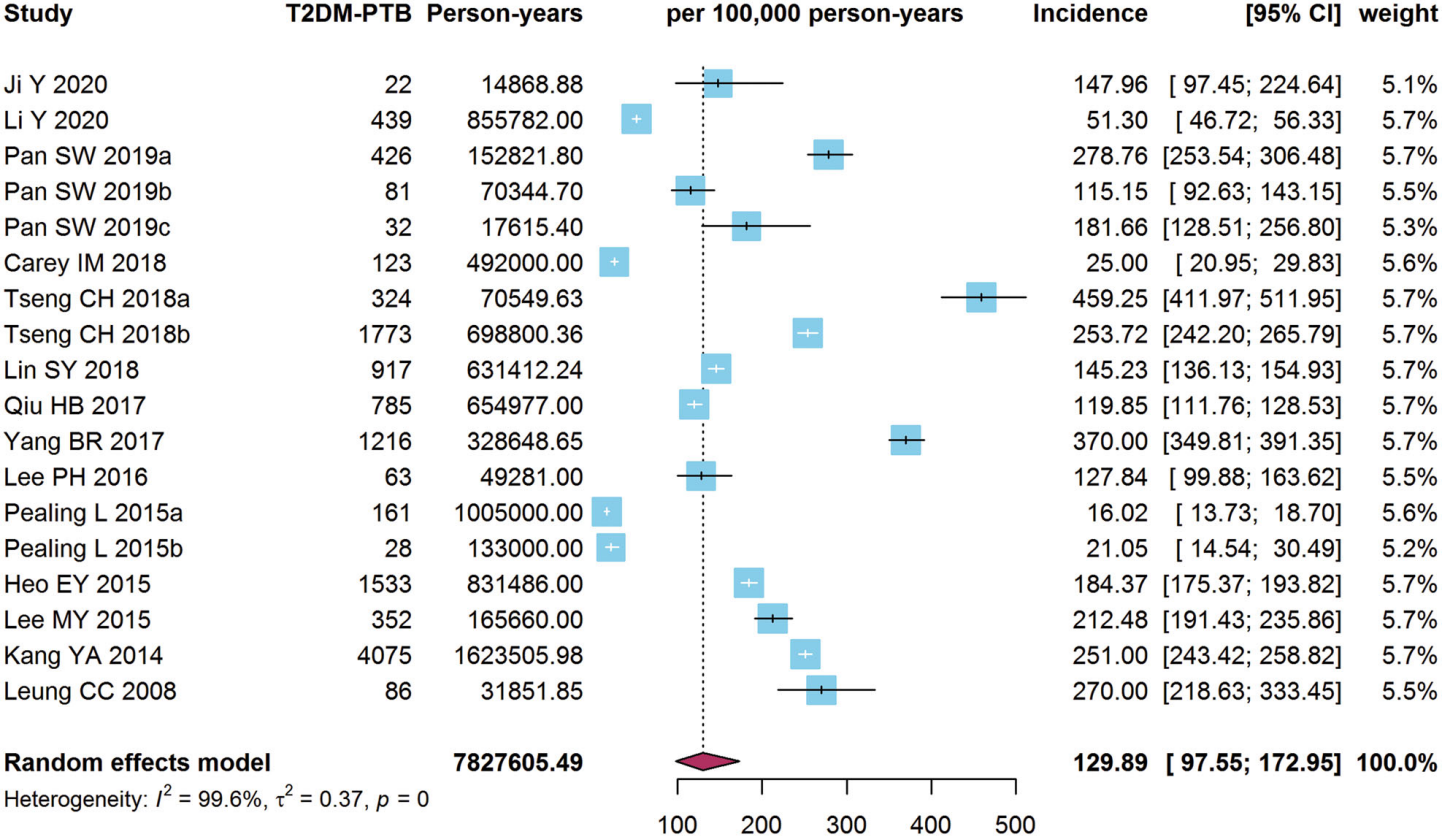
Forest plot of the incidence of pulmonary tuberculosis (PTB) in type 2 diabetes mellitus (T2DM) patients.

Fourteen cohort studies containing 2,533,555 T2DM patients reported the incidence of T2DM-PTB. Since the three studies among them were conducted in different groups, we analysed these studies separately [18,20,25]. The main characteristics of the eligible studies were extracted and listed in Table 1. For significant heterogeneity among the included studies (*I*^2^*=*99.6%, tau^2^=0.37, *p* < 0.001), random effects model was used for incidence pooling. As shown in the forest plot (Figure 2), the overall pooled incidence of PTB among T2DM patients was 129.89 (95% CI: 97.55–172.95) per 100,000 person-years. The T2DM-PTB incidence of these studies ranged from 16.02 (95% CI: 13.73–18.70) to 459.25 (95% CI: 411.97–511.95). Funnel plot of incidence was symmetric visually. The results of Egger’s linear regression test (*t* = −2.06, *p* = 0.06) and Begg’s rank correlation test (*z* = −1.25, *p* = 0.21) showed no significant publication biases (all *p* > 0.05) (Supplementary Figure S1). Sensitivity analysis showed that the estimates after omitting any study did not change significantly from the pooled estimates of T2DM-PTB incidence were robust (Supplementary Figure S2).

**Table 1.** Summary of the studies that reported the PTB incidence in T2DM.

Study	T2DM-PTB (n/N)	Person-years	Period	Country	Region	High TB-burden	Study design	Diagnostic criteria for PTB	QS
Ji Y 2020 [16]	22/14,869	14,868.88	2016–2018	China	Asia	Yes	Cohort	Guidelines for the implementation of China’s tuberculosis control Program (2008)	8
Li Y 2020 [17]	439/240,692	855,782	2010–2015	China	Asia	Yes	Cohort	Guidelines for the implementation of China’s tuberculosis control Program (2008)	8
Pan SW 2019a [18]	426/22,316	152,821.80	2000–2013	Taiwan, China	Asia	Yes	Cohort	ICD-9 codes	7
Pan SW 2019b [18]	81/17,696	70,344.70	2000–2013	Taiwan, China	Asia	Yes	Cohort	ICD-9 codes	7
PanSW 2019c [18]	32/5327	17,615.40	2000–2013	Taiwan, China	Asia	Yes	Cohort	ICD-9 codes	7
Carey IM 2018 [19]	123/96,630	492,000	2008–2015	UK	Europe	No	Cohort	ICD-10 codes	8
Tseng CH 2018a [20]	324/15,799	70,549.63	1999–2011	Taiwan, China	Asia	Yes	Cohort	ICD-9 codes	9
Tseng CH 2018b [20]	1773/148,468	698,800.36	1999–2011	Taiwan, China	Asia	Yes	Cohort	ICD-9 codes	9
Lin SY 2018 [21]	917/49,028	631,412.24	1998–2010	Taiwan, China	Asia	Yes	Cohort	ICD-9 codes	7
Qiu HB 2017 [22]	785/170,399	654,977	2004–2009	China	Asia	Yes	Cohort	Guidelines for the implementation of China’s tuberculosis control Program (2002)	7
Yang BR 2017 [23]	1216/331,601	328,648.65	2009	Korea	Asia	No	Cohort	ICD-10 codes	7
Lee PH 2016 [24]	63/11,260	49,281	2005.3–2009.7	Taiwan, China	Asia	Yes	Cohort	ICD-9 codes	7
Pealing L 2015a [25]	161/190,865	1,005,000	1990–2012	UK	Europe	No	Cohort	ICD-10 codes	8
Pealing L 2015b [25]	28/25,680	133,000	1990–2012	UK	Europe	No	Cohort	ICD-10 codes	8
Heo EY 2015 [26]	1533/331,601	831,486	2009–2011	Korea	Asia	No	Cohort	ICD-10 codes	9
Lee MY 2015 [27]	352/13,981	165,660	1998–2009	Taiwan, China	Asia	Yes	Cohort	ICD-9 codes	7
Kang YA 2014 [28]	4075/840,899	1,623,505.98	2007–2010	Korea	Asia	No	Cohort	ICD-10 codes	7
Leung CC 2008 [39]	86/6444	31,851.85	2000–2005	Hongkong, China	Asia	Yes	Cohort	By reviewing medical or public health records	6

T2DM: type 2 diabetes mellitus; PTB: pulmonary tuberculosis; n/N: number; TB: tuberculosis; QS: quality score; ICD: international Classification of diseases.

### T2DM-PTB prevalence

**Figure 3.**
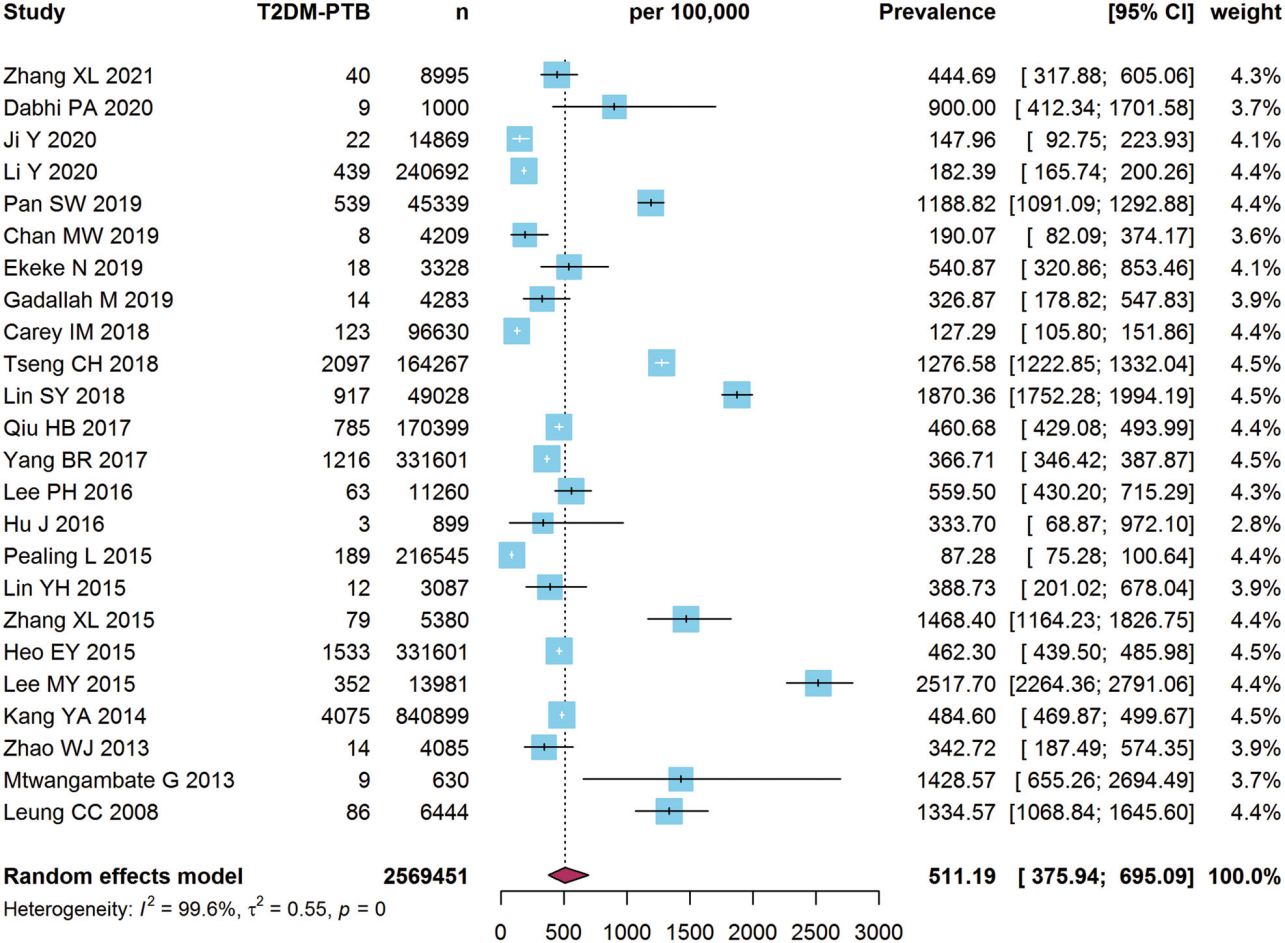
Forest plot of the prevalence of pulmonary tuberculosis (PTB) in type 2 diabetes mellitus (T2DM) patients.

Fourteen cohort studies and ten cross-sectional studies containing 2,569,451 T2DM patients reported the prevalence of T2DM-PTB cases. The main characteristics of the eligible studies were extracted and listed in Table 2. Pooling by random effects model (*I*^2^*=*99.6%, tau^2^ =0.55, *p* <0 .001), the overall pooled prevalence of T2DM-PTB was 511.19 (95% CI: 375.94–695.09) per 100,000 (Figure 3). The T2DM-PTB prevalence of these studies ranged from 87.28 (95% CI: 75.28–100.64) to 2517.70 (95% CI: 2264.36–2791.06). Funnel plot of prevalence was symmetric visually. The results of Egger’s linear regression test (*t* = −0.34, *p* = 0.74) and Begg’s rank correlation test (*z* = 0.30, *p* = 0.77) confirmed no significant publication biases (all *p* > 0.05) (Supplementary Figure S3). In addition, sensitivity analysis indicated that the pooled estimate of T2DM-PTB prevalence was stable (Supplementary Figure S4).

**Table 2.** Summary of the studies that reported the PTB prevalence in T2DM.

Study	PTB	T2DM	Period	Country	Region	High TB-burden	Study design	Risk of bias
Zhang XL 2021 [29]	40	8995	2017.10–2018.9	China	Asia	Yes	Cross-sectional	Moderate
Dabhi PA 2020 [30]	9	1000	2014.9–2015.4	India	Asia	Yes	Cross-sectional	High
Ji Y 2020 [16]	22	14,869	2016–2018	China	Asia	Yes	Cohort	Moderate
Li Y 2020 [17]	439	240,692	2010–2015	China	Asia	Yes	Cohort	Low
Pan SW 2019 [18]	539	45,339	2000–2013	Taiwan, China	Asia	Yes	Cohort	Moderate
Chan MW 2019 [31]	8	4209	2016.2–2016.10	Malaysia	Asia	No	Cross-sectional	High
Ekeke N 2019 [32]	18	3328	2018.2–2018.10	Nigeria	Africa	Yes	Cross-sectional	Moderate
Gadallah M 2019 [33]	14	4283	2012.6–2012.12	Egypt	Africa	No	Cross-sectional	High
Carey IM 2018 [19]	123	96,630	2008–2015	UK	Europe	No	Cohort	Low
Tseng CH 2018 [20]	2097	164,267	1999–2011	Taiwan, China	Asia	Yes	Cohort	Low
Lin SY 2018 [21]	917	49,028	1998–2010	Taiwan, China	Asia	Yes	Cohort	Moderate
Qiu HB 2017 [22]	785	170,399	2004–2009	China	Asia	Yes	Cohort	Moderate
Yang BR 2017 [23]	1216	33,1601	2009	Korea	Asia	No	Cohort	Moderate
Lee PH 2016 [24]	63	11,260	2005.3–2009.7	Taiwan, China	Asia	Yes	Cohort	Moderate
Hu J 2016 [34]	3	899	2013.12–2014.6	China	Asia	Yes	Cross-sectional	Moderate
Pealing L 2015 [25]	189	216,545	1990–2012	UK	Europe	No	Cohort	Low
Lin YH 2015 [35]	12	3087	2012.9–2012.11	Taiwan, China	Asia	Yes	Cross-sectional	Low
Zhang XL 2015 [36]	79	5380	2013	China	Asia	Yes	Cross-sectional	Low
Heo EY 2015 [26]	1533	33,1601	2009–2011	Korea	Asia	No	Cohort	Low
Lee MY 2015 [27]	352	13,981	1998–2009	Taiwan, China	Asia	Yes	Cohort	Moderate
Kang YA 2014 [28]	4075	84,0899	2007–2010	Korea	Asia	No	Cohort	Moderate
Zhao WJ 2013 [37]	14	4085	2012.5–2012.7	China	Asia	Yes	Cross-sectional	Moderate
Mtwangambate G 2013 [38]	9	630	2011.9–2012.3	Tanzania	Africa	Yes	Cross-sectional	High
Leung CC 2008 [39]	86	6444	2000–2005	Hongkong, China	Asia	Yes	Cohort	High

T2DM: type 2 diabetes mellitus; PTB: pulmonary tuberculosis; n/N: number; TB: tuberculosis.

### Subgroup analysis

The results of subgroup analysis are shown in Table 3. The incidence of PTB in patients with T2DM was significantly higher in Asia (187.20 per 100,000 person-years, 95% CI: 147.76–237.17) compared with Europe (20.24 per 100,000 person-years, 95% CI: 14.64–27.99) (*p* < 0.01). A significant higher incidence of T2DM-PTB was observed in countries with a high TB burden (172.04 per 100,000 person-years, 95% CI: 122.98–240.68) than in countries with a low TB burden (73.75 per 100,000 person-years, 95% CI: 40.92–132.91) (*p* = 0.01<0.05). In terms of different criteria for PTB, the incidence of T2DM-PTB was relatively higher in studies by reviewing medical or public health records (270.00 per 100,000 person-years, 95% CI: 218.63–333.45), using International Classification of Diseases (ICD)-9 codes (203.30 per 100,000 person-years, 95% CI: 153.90–268.55), but lower in studies using ICD-10 codes (73.75 per 100,000 person-years, 95% CI: (40.92–132.91) (*p* < 0.01). In addition, the estimated T2DM-PTB incidence was lower in studies whose data collection ended after 2011 (66.18 per 100,000 person-years, 95% CI: 28.73–152.47) than before 2011 (219.81 per 100,000 person-years, 95% CI: 176.15–274.28) (*p* < 0.01).

**Table 3.** Subgroup analysis of PTB incidence and prevalence in T2DM.

Subgroups	*N*	Effect size [95% CI]	*I^2^*	*p* Value of between-subgroup heterogeneity
Incidence (per 100,000 person-years)				
Region				<0.01
Aisa	15	187.20 (147.76, 237.17)	99.3%	
Europe	3	20.24 (14.64, 27.99)	85.7%	
TB-burden				0.01
High	12	172.04 (122.98, 240.68)	99.3%	
Low	6	73.75 (40.92, 132.91)	99.8%	
Criteria of PTB				<0.01
ICD-9	8	203.30 (153.90, 268.55)	98.4%	
ICD-10	6	73.75 (40.92, 132.91)	99.8%	
Guidelines for the implementation of China’s TB control Program (2008)	2	85.37 (30.25, 240.87)	95.8%	
Guidelines for the implementation of China’s TB control Program (2002)	1	119.85 (111.76, 128.53)	–	
By reviewing medical or public health records	1	270.00 (218.63, 333.45)	–	
Ending year of data collection				<0.01
≤2011	10	219.81 (176.15, 274.28)	99.2%	
>2011	8	66.18 (28.73, 152.47)	99.5%	
Prevalence (per 100,000)				
Region				<0.01
Aisa	19	594.19 (431.86–817.54)	99.6%	
Africa	3	617.43 (287.07, 1327.96)	83.5%	
Europe	2	105.01 (72.55, 151.98)	90.6%	
TB-burden				<0.01
High	17	692.15 (468.75, 1022.04)	99.4%	
Low	7	250.73 (180.22, 348.84)	99.2%	
Study design				0.89
Cohort	14	503.19 (339.72, 745.30)	99.8%	
Cross-sectional	10	524.95 (324.17, 850.07)	90.4%	

PTB: pulmonary tuberculosis; TB: tuberculosis; N: number, CI: confidence intervals.

The prevalence of T2DM-PTB was also significantly higher in countries with a high TB burden (692.15 per 100,000, 95% CI: 468.75–1022.04) than that in countries with a low TB burden (250.73 per 100,000, 95% CI: 180.22–348.84) (*p* < 0 .01). Besides, the prevalence of T2DM-PTB was significantly higher in Asia (594.19 per 100,000, 95% CI: 431.86–817.54) and Africa (617.43 per 100,000, 95% CI: 287.07–1327.96) than in Europe (105.01 per 100,000, 95% CI: 72.55–151.98) (*p* < 0 .01). However, no significant difference in the prevalence of T2DM-PTB was found when stratified according to study design (*p* = .89).

## Discussion

This review quantitatively synthesized the existing evidence based on 24 studies involving a total of 2,569,451 T2DM patients, and revealed that the estimated overall incidence and prevalence of PTB among T2DM patients were 129.89 (95% CI: 97.55–172.95) per 100,000 person-years and 511.19 (95% CI: 375.94–695.09) per 100,000 respectively. The mechanisms that explain the association between T2DM and increased susceptibility to PTB are barely understood. One of the main causes for the aggravation and even death of T2DM patients is their high susceptibility to many infectious diseases including PTB, which may be related to immune dysfunction [40]. In terms of the high incidence and prevalence of T2DM-PTB, preventive interventions among T2DM patients are imperative.

It is worth noting that the incidence of PTB in T2DM patients varies significantly in different geographical regions, which was significantly higher in Asia countries than that in Europe countries. This is in accordance with the finding reported in another systematic review that DM patients in Asian continent (2.46, 95% CI: 2.04–3.02) were at higher risk of TB than DM patients in Europe (1.71, 95% CI: 1.33–2.20). The incidence of T2DM-PTB was significant higher in countries with a high TB burden than in countries with a low TB burden, which may be related to the socio-economic conditions and access to medical healthcare. It highlights the need to implement prevention interventions, such as T2DM-PTB bidirectional screening, to reduce the dual burden especially in low-income and middle-income countries. The literatures included in this meta-analysis have different criteria for PTB diagnosis. According to the subgroup analysis, the incidence of T2DM-PTB differed between subgroups. Our results showed that the T2DM-PTB incidence was higher in studies by reviewing medical or public health records and using ICD-9 codes. It indicated that different methods may affect the results of PTB diagnosis in T2DM patients. The first study to complete data collection ended in 2005 [39], but the latest study to complete data collection ended in 2018 [16]. In terms of the ending year of data collection, we found the estimated T2DM-PTB incidence was significantly lower for studies whose data collection ended after 2011 than before 2011. It can be speculated that the decline in T2DM-PTB incidence may be is associated with substantial progress made in the areas of TB prevention, diagnosis and treatment.

The pooled prevalence of T2DM-PTB was much lower than the prevalence of DM-TB (4.72%, 95% CI: 3.62–5.83%) reported in a previous meta-analysis [41], which might be attributed to different study population and inclusion criteria. Our study found significant higher prevalence of T2DM-PTB in Africa and Asia, which consistent with findings reported by previous meta-analyses [41,42]. It might partly be attributable to the fact that most of the studies included in this meta-analysis were conducted in endemic regions. When evaluated by TB burden of study countries, the prevalence of T2DM-PTB appeared to be higher in countries with a high TB burden compared to countries with a low TB burden. This finding is supported by the fact that the number of new TB cases worldwide is mostly from countries with a high TB burden, such as India, China and Nigeria. In terms of study design, there was no significant difference in the prevalence of T2DM-PTB between cohort studies and cross-sectional studies.

There are several limitations should be acknowledged in this meta-analysis. First of all, there has been epidemiological evidence that age [43], the status of glycemic control [44], cigarette smoking [41], gender and body mass index [37] are factors related to the prevalence of TB among DM patients. However, in most of included studies lack of information about these variables, restricting our ability to identify specific traits in subgroup analyses. Second, the heterogeneity among the included studies was significant, which may be caused by unmeasured characteristics. Thus, the pooled incidence and prevalence need to be interpreted with caution. Third, most of these studies in this meta-analysis were cross-sectional and did not set up a control group, so there was a lack of reasonable comparison. Fourth, our comprehensive search strategy has identified many eligible studies, but the majority of them conducted in Asia. As we known, most of the 30 countries with high-TB burden were from Asia and Africa. In countries with low-TB burden, there is little research in the field of T2DM-PTB comorbidity. To some extent, it might have implications for the precision and representativeness of the estimates.

Despite these limitations, to the best of our knowledge, this is the first meta-analysis that quantitatively summarizes existing evidence about the global incidence and prevalence of PTB in patients with T2DM from a large number of studies. The findings of our study shed light on the impact of T2DM in the development of PTB, and can be further used for performing cost-effectiveness analysis and cost-utility analysis of screening programmes for PTB among T2DM patients.

## Conclusions

Taken together, our findings provide a theoretical basis for the necessity of PTB screening in T2DM patients and implementation of effective prevention programs to mitigate the dual burden of PTB-T2DM co-morbidity, particularly in countries with a high TB burden. The mechanism by which T2DM increases the risk of PTB at the individual and population levels are complex and still needs to be further clarified.

## Supplementary Material

Supplemental Material

## Data Availability

Data sharing is not applicable to this article, as this study is a meta-analysis.
