# Supplementary material for: Incidence and prevalence of pulmonary tuberculosis among patients with type 2 diabetes mellitus: a systematic review and meta-analysis
Source: Ann Med. 2022 Jun 15;54(1):1657–66. doi: 10.1080/07853890.2022.2085318 (PMC9225779; doi:10.1080/07853890.2022.2085318)
Supplement: Supplemental Material [file IANN_A_2085318_SM0313.doc]

**Table S1. The list of the 43 studies that were eventually excluded.**

| Number | Title |
| --- | --- |
| [1] | Screening of patients with tuberculosis for diabetes mellitus in India, Tropical medicine & international health : TM & IH 18 (2013) 636-45. |
| [2] | B. Abdelmoez, A. Abd-El-Nasser, M.G. Baheeg and A.A. Sedky, Prevalence of tuberculosis among children who had type 1 diabetes and were admitted to Elminia University Hospital, Pediatrics 121 (2008) S151-S151. |
| [3] | M. Abdulrazaq and A. Alfarttoosi, Prevalence of diabetes mellitus in new tuberculosis cases in Baghdad, European Respiratory Journal 46 (2015). |
| [4] | A. Abera and G. Ameya, Pulmonary Tuberculosis and Associated Factors Among Diabetic Patients Attending Hawassa Adare Hospital, Southern Ethiopia, The open microbiology journal 12 (2018) 333-342. |
| [5] | M. Ahmed, I. Omer, S.M.A. Osman and E.H. Ahmed-Abakur, Association between Pulmonary Tuberculosis and Type 2 Diabetes in Sudanese Patients, International journal of mycobacteriology 6 (2017) 97-101. |
| [6] | L. Airaghi and A. Tedeschi, Negative association between occurrence of type 1 diabetes and tuberculosis incidence at population level, Acta diabetologica 43 (2006) 43-5. |
| [7] | B. Alisjahbana, S.M. McAllister, C. Ugarte-Gil, N.M. Panduru, K. Ronacher, R.C. Koesoemadinata, C. Zubiate, A.L. Riza, S.T. Malherbe, L. Kleynhans, S. Lopez, H.M. Dockrell, R. Ruslami, M. Ioana, G. Walzl, F. Pearson, J.A. Critchley, D.A.J. Moore, R. van Crevel and P.C. Hill, Screening diabetes mellitus patients for pulmonary tuberculosis: a multisite study in Indonesia, Peru, Romania and South Africa, Transactions of the Royal Society of Tropical Medicine and Hygiene 115 (2021) 634-643. |
| [8] | B. Alisjahbana, E. Sahiratmadja, E.J. Nelwan, A.M. Purwa, Y. Ahmad, T.H. Ottenhoff, R.H. Nelwan, I. Parwati, J.W. van der Meer and R. van Crevel, The effect of type 2 diabetes mellitus on the presentation and treatment response of pulmonary tuberculosis, Clinical infectious diseases : an official publication of the Infectious Diseases Society of America 45 (2007) 428-35. |
| [9] | L.R. Armstrong, J.S. Kammerer and M.B. Haddad, Diabetes mellitus among adults with tuberculosis in the USA, 2010-2017, BMJ open diabetes research & care 8 (2020). |
| [10] | R. Becker and K. Seige, Incidence, age pattern and course of tuberculosis in diabetes mellitus, Zeitschrift fur Alternsforschung 9 (1955) 46-55. |
| [11] | N. Berkowitz, A. Okorie, R. Goliath, N. Levitt, R.J. Wilkinson and T. Oni, The prevalence and determinants of active tuberculosis among diabetes patients in Cape Town, South Africa, a high HIV/TB burden setting, Diabetes research and clinical practice 138 (2018) 16-25. |
| [12] | J. Cai, A. Ma, Y. Wang, Q. Wang, X. Han, S. Zhao and Y. Ma, The prevalence and associated factors of diabetes in pulmonary tuberculosis patients in rural China, Faseb Journal 29 (2015). |
| [13] | M. Castellanos-Joya, G. Delgado-Sanchez, L. Ferreyra-Reyes, P. Cruz-Hervert, E. Ferreira-Guerrero, G. Ortiz-Solis, M. Irene Jimenez, L. Lorena Salazar, R. Montero-Campos, N. Mongua-Rodriguez, R. Baez-Saldana, M. Bobadilla-del-Valle, J. Felipe Gonzalez-Roldan, A. Ponce-de-Leon, J. Sifuentes-Osornio and L. Garcia-Garcia, Results of the Implementation of a Pilot Model for the Bidirectional Screening and Joint Management of Patients with Pulmonary Tuberculosis and Diabetes Mellitus in Mexico, PloS one 9 (2014). |
| [14] | V.P. Chukanova, A.S. Sergeev, L.E. Pospelov and A.L. Sobkin, Epidemiological and immunogenetic analysis of tuberculosis and diabetes mellitus association, Problemy tuberkuleza (2000) 11-4. |
| [15] | B.M. Cohen, Diabetes mellitus among Indians of the American Southwest: its prevalence and clinical characteristics in a hospitalized population, Annals of internal medicine 40 (1954) 588-99. |
| [16] | J. Cordeiro da Costa, O. Oliveira, L. Baía, R. Gaio, M. Correia-Neves and R. Duarte, Prevalence and factors associated with diabetes mellitus among tuberculosis patients: a nationwide cohort, The European respiratory journal 48 (2016) 264-8. |
| [17] | C.C. Dobler, J.R. Flack and G.B. Marks, Risk of tuberculosis among people with diabetes mellitus: an Australian nationwide cohort study, BMJ open 2 (2012) e000666. |
| [18] | R.F. Dyck, H. Klomp, D.D. Marciniuk, L. Tan, M.R. Stang, H.A. Ward and V.H. Hoeppner, The relationship between diabetes and tuberculosis in Saskatchewan - Comparison of registered Indians and other Saskatchewan people, Canadian Journal of Public Health-Revue Canadienne De Sante Publique 98 (2007) 55-59. |
| [19] | K.C. Erisa, K.O. Robsky, P.J. Kitonsa, A. Nalutaaya, D. Isooba, O. Nakasolya, J. Mukiibi, D. Dowdy, E.A. Kendall and A. Katamba, Low prevalence of diabetes mellitus in TB patients and the community in urban Uganda, International Journal of Tuberculosis and Lung Disease 25 (2020) 590-592. |
| [20] | C.P. Fu, C.L. Lee, Y.H. Li and S.Y. Lin, Metformin as a potential protective therapy against tuberculosis in patients with diabetes mellitus: A retrospective cohort study in a single teaching hospital, Journal of diabetes investigation 12 (2021) 1603-1609. |
| [21] | M.C.E. Garcia Sancho, M.D.J. Castillejos Lopez and M.G.F. San Miguel, Impact of type 2 diabetes on the prevalence of respiratory diseases in a third level hospital, Diabetes 56 (2007) A622-A622. |
| [22] | M. Gedfew, M. Ayana, A. Abate, B. Bewket, D. Haile, A. Edmealem and A. Andualem, Incidence and Predictors of Tuberculosis among Adult Diabetic Patients, Debre Markos Referral Hospital, Northwest Ethiopia, 2018: A Retrospective Cohort Study, Diabetes, metabolic syndrome and obesity : targets and therapy 13 (2020) 869-878. |
| [23] | J.E. Golub, Y. Mok, S. Hong, K.J. Jung, S.H. Jee and J.M. Samet, Diabetes mellitus and tuberculosis in Korean adults: impact on tuberculosis incidence, recurrence and mortality, International Journal of Tuberculosis and Lung Disease 23 (2019) 507-+. |
| [24] | A. Jabbar, S.F. Hussain and A.A. Khan, Clinical characteristics of pulmonary tuberculosis in adult Pakistani patients with co-existing diabetes mellitus, Eastern Mediterranean health journal = La revue de sante de la Mediterranee orientale = al-Majallah al-sihhiyah li-sharq al-mutawassit 12 (2006) 522-7. |
| [25] | M.V. Jali, V.K. Mahishale and M.B. Hiremath, Bidirectional screening of tuberculosis patients for diabetes mellitus and diabetes patients for tuberculosis, Diabetes & metabolism journal 37 (2013) 291-5. |
| [26] | L.S. Kartoziia and T.P. Kordzakhiia, Pulmonary tuberculosis in patients with diabetes mellitus according to data from a tuberculosis hospital, Problemy tuberkuleza 52 (1974) 54-6. |
| [27] | S.J. Kim, Y.P. Hong, W.J. Lew, S.C. Yang and E.G. Lee, Incidence of pulmonary tuberculosis among diabetics, Tubercle and lung disease : the official journal of the International Union against Tuberculosis and Lung Disease 76 (1995) 529-33. |
| [28] | I.E. Kossii, M.A. Karachunskii, G.O. Kaminskaia, N.A. Chernykh and D.E. Zhukovskaia, Pulmonary tuberculosis in patients with different types of diabetes mellitus, Problemy tuberkuleza (2002) 21-4. |
| [29] | S. Kumpatla, A. Sekar, S. Achanta, B.N. Sharath, A.M.V. Kumar, A.D. Harries and V. Viswanathan, Characteristics of patients with diabetes screened for tuberculosis in a tertiary care hospital in South India, Public health action 3 (2013) S23-8. |
| [30] | E.H. Lee, J.M. Lee, Y.A. Kang, A.Y. Leem, E.Y. Kim, J.Y. Jung, M.S. Park, Y.S. Kim, S.K. Kim, J. Chang and S.Y. Kim, Prevalence and Impact of Diabetes Mellitus Among Patients with Active Pulmonary Tuberculosis in South Korea, Lung 195 (2017) 209-215. |
| [31] | T.H. Lee and S.H. Han, The prevalence of complications in Korean diabetic subjects, The Tohoku journal of experimental medicine 141 Suppl (1983) 361-5. |
| [32] | Y. Lin, L. Li, F. Mi, J. Du, Y. Dong, Z. Li, W. Qi, X. Zhao, Y. Cui, F. Hou, R. Zachariah, A. Kapur, K. Loennroth and A.D. Harries, Screening patients with Diabetes Mellitus for Tuberculosis in China, Tropical Medicine & International Health 17 (2012) 1302-1308. |
| [33] | V. Mave, S. Nimkar, H. Prasad, D. Kadam, S. Meshram, R. Lokhande, N. Gupte, D. Jain, A. Gupta and J.E. Golub, Tuberculosis screening among persons with diabetes mellitus in Pune, India, BMC infectious diseases 17 (2017) 388. |
| [34] | S.M. McAllister, R.C. Koesoemadinata, P. Santoso, N.N.M. Soetedjo, A. Kamil, H. Permana, R. Ruslami, J.A. Critchley, R. van Crevel, P.C. Hill and B. Alisjahbana, High tuberculosis incidence among people living with diabetes in Indonesia, Transactions of the Royal Society of Tropical Medicine and Hygiene 114 (2020) 79-85. |
| [35] | A.K. Pannu, A. Saroch, V. Singla, N. Sharma, P. Dutta, A. Jain and A. Angrup, Clinical spectrum, etiology and outcome of infectious disease emergencies in adult diabetic patients in northern India, Diabetes & metabolic syndrome 14 (2020) 921-925. |
| [36] | P.S. Reddy, A. Mohan, B.S. Kumar, A. Sachan and D.P. Kumar, Screening for tuberculosis in 1,005 adult patients with diabetes mellitus at a tertiary care hospital, The Journal of the Association of Physicians of India 64 (2016) 115-115. |
| [37] | B.I. Restrepo, S.P. Fisher-Hoch, J.G. Crespo, E. Whitney, A. Perez, B. Smith and J.B. McCormick, Type 2 diabetes and tuberculosis in a dynamic bi-national border population, Epidemiology and infection 135 (2007) 483-91. |
| [38] | A. Sil, D. Patra, P. Dhillon and P. Narasimhan, Co-existence of diabetes and TB among adults in India: a study based on National Family Health Survey data, Journal of biosocial science 53 (2021) 758-772. |
| [39] | C. Ugarte-Gil, M. Curisinche, E. Herrera-Flores, H. Hernandez and J. Rios, Situation of the tuberculosis-diabetes comorbidity in adults in Peru: 2016-2018, Revista peruana de medicina experimental y salud publica 38 (2021) 254-260. |
| [40] | I.M. Voloshyn, S.G. Podhaievs'kyi and V.F. Kovalenchenko, Clinical course, diagnosis and specific features of treatment of pulmonary tuberculoma in patients with diabetes mellitus, Klinichna khirurhiia (1999) 25-7. |
| [41] | S. Wafy, Presentations and treatment response of pulmonary tuberculosis in type 2 diabetes mellitus, European Respiratory Journal 40 (2012). |
| [42] | R.J. Wilkinson, Tuberculosis and Type 2 Diabetes Mellitus: An Inflammatory Danger Signal in the Time of Coronavirus Disease 2019, Clinical infectious diseases : an official publication of the Infectious Diseases Society of America 72 (2021) 79-81. |
| [43] | A.B. Swai, D.G. McLarty and F. Mugusi, Tuberculosis in diabetic patients in Tanzania, Tropical doctor 20 (1990) 147-50. |

**
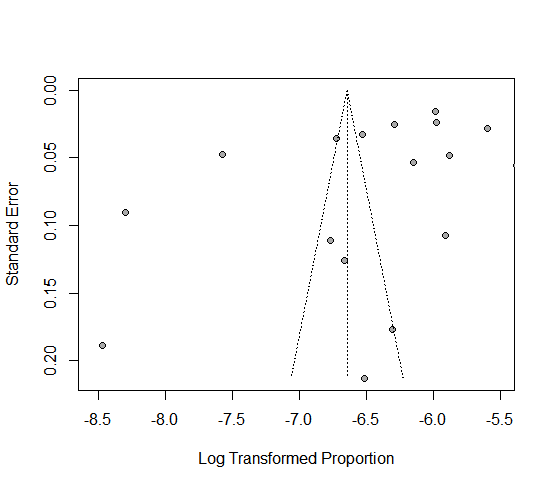
**

**Figure S1** Funnel plot of the T2DM-PTB incidence studies.

**
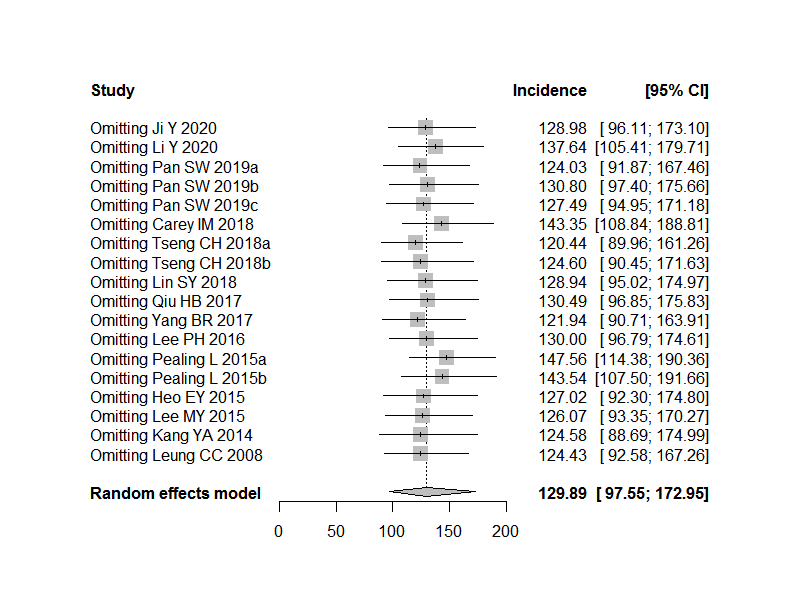
**

**Figure S2** Sensitivity analyses for the T2DM-PTB incidence studies.

**
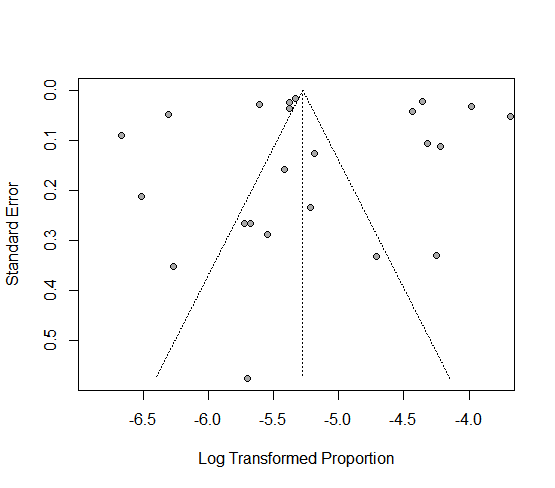
**

**Figure S3** Funnel plot of the T2DM-PTB prevalence studies.

**
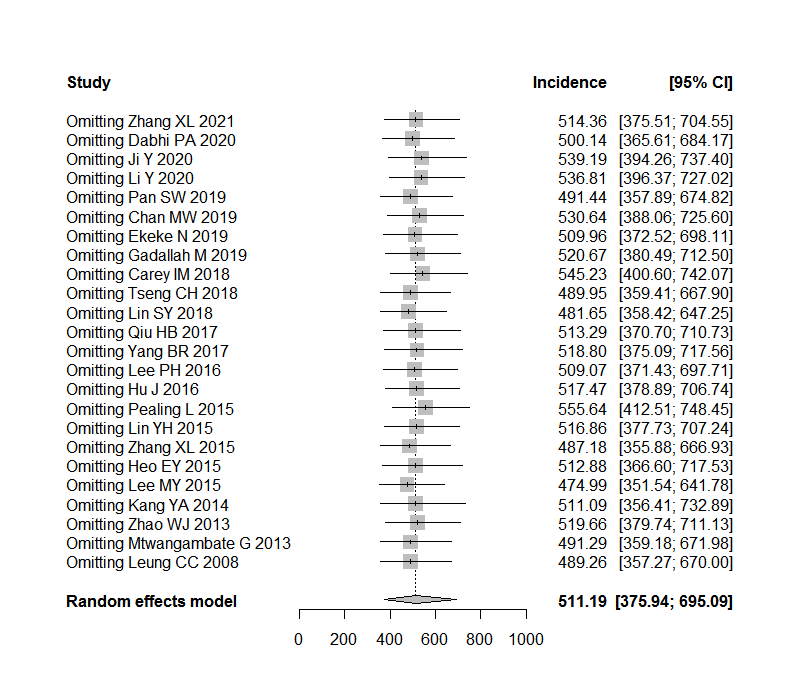
**

**Figure S4** Sensitivity analyses for the T2DM-PTB prevalence studies.
